# The role of psychiatrists in diagnosing conversion disorder: a mixed-methods analysis

**DOI:** 10.2147/NDT.S96330

**Published:** 2016-05-11

**Authors:** Richard A Kanaan, David Armstrong, Simon Wessely

**Affiliations:** 1 Department of Psychiatry, University of Melbourne, Austin Health, Heidelberg, VIC, Australia; 2 Department of Psychological Medicine, King’s College London, Institute of Psychiatry, Weston Education Centre, Denmark Hill, London, UK; 3 Department of General Practice, King’s College London, Capital House, London, UK

**Keywords:** functional neurological disorders, classification, qualitative research, survey, psychiatric formulation

## Abstract

**Objective:**

Since DSM-5 removed the requirement for a psychosocial formulation, neurologists have been able to make the diagnosis of conversion disorder without psychiatric input. We sought to examine whether neurologists and specialist psychiatrists concurred with this approach.

**Design:**

We used mixed methods, first surveying all the neurologists in the UK and then interviewing the neuropsychiatrists in a large UK region on the role of psychiatrists in diagnosing conversion disorder.

**Results:**

Of the surveyed neurologists, 76% did not think that psychiatrists were essential for the diagnosis and 71% thought that psychiatrists did not even consider conversion disorder when referred a case. The neuropsychiatrists who were interviewed held complex models of conversion disorder. They believed all cases could be explained psychosocially in theory, but the nature of the diagnostic encounter often prevented it in practice; all felt that psychosocial formulation could be very helpful and some felt that it was essential to diagnosis.

**Conclusion:**

Although neurologists do not think psychiatrists are required for diagnosing conversion disorder, specialist psychiatrists disagree, at least in some cases.

## Introduction

With the advent of the *Diagnostic and Statistical Manual of Mental Disorders*, *fifth edition* (DSM-5),^1^ the diagnosis of conversion disorder no longer requires a psychosocial formulation. This means that neurologists can now realistically diagnose conversion disorder without a psychiatrist.^2^,^3^ Although neurologists are fairly confident that they can make the diagnosis^4^ and feel that psychiatrists are usually of little help,^5^ the prospect of actually diagnosing may fill them with apprehension.^6^

Although these are changes to criteria, not conceptualization,^3^ by criteria alone DSM-5 now presents conversion disorder as an unexplained neurological disorder, without any obvious psychogenic element^7^ – and changes to conceptualization may well follow. Do psychiatrists concur with this? Do they add anything to neurologists’ diagnoses? Do they think they add anything?

The aim of this study is to explore the views of neurologists and neuropsychiatrists on the need for psychiatric input in diagnosing conversion disorder.

## Methods

This study employed mixed methods: a quantitative survey of all consultant neurologists in the UK, which guided a qualitative exploration of the understanding and diagnosis of conversion disorder in a group of consultant neuropsychiatrists. King’s College Hospital Research Ethics Committee approved the study. A response was taken to indicate consent for the survey; all interviewees gave written, informed consent.

### Survey

A postal survey was sent to all consultant neurologists registered with the Association of British Neurologists, with a follow-up sent to nonrespondents after 4 weeks. The survey contained 33 largely multiple-choice questions exploring their understanding and management of conversion disorder. Survey questions 19 and 20 addressed the role of psychiatrists in the diagnostic process (Table 1). Further details, including previously reported elements and a copy of the full survey, are in the studies by Kanaan et al,^4^,^8^ and Wojcik et al.^9^

### Interviews

All practising consultant neuropsychiatrists (fully qualified specialists in the psychiatry of neurology) in a large UK region were invited to interview by RAK, with further recruitment by snowball sampling. Respondents had depth interviews conducted by RAK in their own offices. Interviews followed a broad topic guide, exploring their understanding of conversion disorder, its diagnosis and management. Interviews were audio recorded, transcribed, coded, and inductively analyzed, consistent with grounded theory, using NVIVO 10 software. The methodology was previously employed in interviews with neurologists in the region, described here.^6^,^8^,^10^

## Results

### Survey

Of the 616 neurologists surveyed, 319 responded in the first round and 57 in the second round: excluding wrong addresses, blank responses, and those who are not currently practicing, gave an adjusted response rate of 349 from 591 eligible subjects (59%). The majority of respondents were male (82%), median age range 46–50, with mean 20 years of neurology experience.

Only a minority thought a psychiatric opinion essential for diagnosis, and the vast majority thought that psychiatrists routinely failed to even look for conversion disorder when patients were referred to them (Table 1). Fifteen neurologists selected “Other” for Q20, but their comments largely seemed like nuanced versions of the listed options (eg, “I may have got the wrong diagnosis and would reassess,” “psychiatrist is not interested”), though others suggested their interpretation would vary depending on the psychiatrist or that they would instead seek a psychological opinion.

### Interviews

Ten neuropsychiatrists were interviewed. Seven were male, their median age was 48, and had mean 13 months of neurology training prior to entering psychiatry. They included all but one of the neuropsychiatrists in the region plus three from a tertiary-referral center just outside the region as led by the snowball sampling.

All of the subjects reported seeing a great deal of conversion disorder, and all engaged carefully with the question of its nature. They saw the area as highly complex and employed a remarkably wide range of models in their analysis. Models such as self-deception, somatization, dissociation, conversion, social causation, social construction, cognitive bias, and feigning were employed but thought too simplistic alone, and all respondents used more than one.

… thought to be a conversion of the reaction to that trauma into the physical symptom. However … it actually is a much murkier area, because it overlaps with other … concepts such as somatisation, and even factitious behaviours … and illness behaviour … [S1]

Well, it varies … from patient to patient … some sort of catastrophic reaction to a probable genuine medical event … and this has given rise to … catastrophic thinking about their health, and has been reinforced by seeing lots of medical doctors and having lots of tests, and people saying they don’t know what it is but not telling them they think it might be … [S5]

I think in most cases … it’s a reaction to some kind of difficulty in life … what happens is that the … abnormal illness behaviour … at some level, even if it is at an unconscious level, this is quite appealing to people … and I think there’s also … a very important element of self deception. [S7]

All thought the condition was, broadly speaking, psychogenic, and all thought they could formulate (ie, provide a psychosocial explanation for the patient’s illness) at least some of the time, although in terms of the complex array of models listed earlier:

I think Freudian ideas are actually quite relevant, but … it’s wrong to sometimes impute unconscious motives to the patient when there isn’t direct evidence for them. And I’m less convinced by whatever symbolic representation the symptom has in terms of primary gain … than the wider social and cultural influences, and personal knowledge, and all those other things that actually feed into the particular symptom at the particular time. [S2]

Where they could not find a clear psychosocial explanation, they held that a formulation was there to be discovered and if it was not found, this represented an unavoidable feature of the disorder or its investigation:

… the symptoms serve … a psychological purpose, which could be construed as a form of defence … And the failure of a patient to come up with their own psychological formulation is not surprising in terms of that defence. [S3]

… partly I think that is because of the psychological, almost adversarial, set that many somatoform patients, particularly those who’ve got a long history of … shopping around for diagnosis … they’ll often be very defensive, perhaps understandably so. [S8]

Accordingly, given the uncertainty around formulation, the diagnostic relationship with neurologists was also complex and varied with the certainty of the neurological assessment. Some saw a role in acting as a kind of second opinion, and most that a positive formulation would be diagnostically important where there was doubt:

… there is a role in terms of confirming diagnosis … because in some cases … despite the investigations it can be unclear … and … doing the psychiatric assessment … you may uncover … the psychological issues that … explain some of the symptoms. [S6]

For others, if they could not formulate, they would not make the diagnosis of conversion disorder:

If I don’t come up with a formulation that’s convincing for me, then I can’t assign any of those somatoform diagnoses … and much to the dislike of the neurologists, I simply write back and say that the patient has medically unexplained symptoms of which we have no psychological basis at present to diagnose a psychogenic condition … I only make the diagnosis, not as a diagnosis of exclusion, but … where there is evidence to back it up. [S10]

## Discussion

These results confirmed the view that most neurologists do not see psychiatrists as essential and in many cases see them as unhelpful to the diagnostic process in conversion disorder: the commonly reported experience of rejected psychiatric referrals^5^ reflecting psychiatrists’ failure to even consider the diagnosis in their assessments. This suggests that many neurologists face a practical imperative to diagnose conversion disorder solely from a neurological perspective: they cannot rely on obstructive psychiatrists for the diagnosis of the commonest single condition referred to their outpatient clinics.^11^

The interviews with the neuropsychiatric specialists revealed a different perspective, regarding their role in psychiatric formulation as not only helpful but also, for some at least, essential. By contrast with the neurologists previously interviewed, who typically felt they did not need to understand it, and were happy to accept “textbook” accounts on trust,^10^ the neuropsychiatrists had highly developed, closely held views on the nature of conversion disorder and were comfortable with the complexity its formulation required. Furthermore, all felt that it was psychogenic.

Although neurologists may not think that psychiatrists have a sufficient model for conversion disorder,^4^ this study suggests that psychiatrists think they do – at least some, and in some cases – and that excluding that perspective would lead to misdiagnosis. Of course, those we interviewed were specialists in neuropsychiatry and probably not the kind of psychiatrists neurologists find unhelpful (our previous interviews with the neurologists in the same region revealed great support for their neuropsychiatric liaisons) and definitely not representative of psychiatrists in general, even those working in consultation-liaison. On this evidence, if there is an argument to be made from the failure of psychiatry to fulfill its role in the current diagnostic scheme, perhaps it is not that psychiatrists should be excluded from the process but that their training in conversion disorder should be improved, so they may respond more appropriately to neurological referral.

This study is, inevitably, limited in a number of ways. The study investigated only UK doctors; the response rates may have introduced a degree of selection bias; and responses may have been shaped by the structure of the survey and the nature of the interview (in particular, that all interviewees were known professionally to the interviewer). Finally, it was conducted at a time when DSM-IV was still in force, so interviewees may have felt that formulation was the necessity that manual required.

## Figures and Tables

**Table 1:** Neurologists’ views on psychiatrists’ diagnostic role in conversion disorder

19. What role do psychiatrists have in the diagnosis of conversion disorder? (n=343)	
Essential	81 (24%)
Helpful, but not necessary	145 (42%)
Not helpful	117 (34%)
20. If a psychiatrist sends a patient you referred with conversion back to you saying they can find no psychiatric disorder, what would you presume? (n=347)	
I must have been wrong about conversion and should look again	25 (7%)
They have only excluded other psychiatric disorders, not conversion	245 (71%)
They have looked for conversion, but failed to find it	38 (11%)
Combination of the above	8 (2%)
Other	31 (9%)

**Notes:** Questions 19 and 20 are from a postal survey seeking neurologists views on the diagnosis and management of conversion disorder. The numbers represent those endorsing each option.
